# The Degree of Meeting the Needs of Older People with Frailty Syndrome in the Residential Environment in Relation to Interventions—Experimental Study

**DOI:** 10.3390/ijerph191811682

**Published:** 2022-09-16

**Authors:** Aneta Soll-Morka, Donata Kurpas

**Affiliations:** 1 Institute of Health Sciences, University of Opole, 45-040 Opole, Poland; 2 Department of Family Medicine, Wroclaw Medical University, 50-367 Wrocław, Poland

**Keywords:** aging, frailty syndrome, diet, physical activity, older adults, unmet needs

## Abstract

The study aimed to determine the degree of satisfaction with health, psychological, environmental, and social needs and to determine the effects of a nutritional intervention, physical activity, and comprehensive activity (nutritional intervention plus physical activity) on the degree of satisfaction of the needs of older people with frailty syndrome (FS). The study included 188 residents (140 women and 48 men) and was conducted using the Fried scale and Camberwell’s modified brief needs assessment. In addition, data were collected on age, sex, educational level, type of the previous occupation, marital status, remaining in a relationship, co-residents, place of residence, work status, financial situation, and help with housework. Intervention groups were formed: G1-diet, G2-physical activity, G3-comprehensive therapy, and G4-control. Stage 1 (T1)-3 months after the first examination, stage 2 (T2)-after another three months, the measurements from stage 0. In all groups, the majority were women, respondents with a low or medium level of education in relationships. The degree of need satisfaction in groups G2, G3, and G4 depended on the measurement time (*p* = 0.019, *p* = 0.007, *p* = 0.016). The introduction of physical activity and physical activity in combination with dietary changes most effectively influenced the increase in the level of need satisfaction in elderly patients with frailty.

## 1. Introduction

The number of older adults in Poland and around the world is associated with many adverse medical, social, economic, and other problems. The reduction or loss of functional, physical, mental, and cognitive ability increases the need for medical and social care [1,2]. Demographic aging is also expanding the number of people with frailty [3,4].

Frailty is a dynamic condition and contains physical, psychological, and social factors that interact with each other, disturb the body’s homeostasis of the organism and lead to negative consequences. This multifaceted approach seems appropriate for evaluating this problem in care planning [5,6,7]. From a clinical and physical point of view, the factors related to the aging of the body, such as a decrease in muscle strength, a decrease in lean body mass, a deterioration in balance, a decrease in endurance, a decrease in the ability to move as well as a reduction in physical activity, are considered factors in the development of the frailty. The presence of several examined features indicates frailty [8]. The assessment of mood, cognitive functions, and nutritional status is also important [3]. Research in the world shows that frailty, considered in a broad aspect, more often affects women, people with lower education and a lower economic standard of living, increases with age and is associated with chronic diseases and disabilities [9]. The consequence is an increase in the number of potential needs in this group of patients that should be met by health and social care [10].

The satisfied and unmet needs of older adults significantly affect their quality of life and the entire treatment process. Assessing patients’ needs is essential for planning the scope of therapeutic and social management. The most beneficial is personalized care adapted to older adults’ needs, which are complex and often long-term due to the coexistence of disability, physical and mental illnesses, and social problems [11].

Older adults face many physical, social, and psychological challenges associated with chronic diseases in three main areas: activity and social relationships, mental health, and activities related to mobility, self-care, and home life [12]. The factors determining and modifying the level of satisfied or unmet needs of patients are multiple morbidities, physical limitations, sociodemographic features (age, gender, income level, education level), quality of communication between the patient and the service provider, access to primary health care, availability of specialist care, and type of insurance [13]. The level of satisfying needs is also influenced by proficiency in complex activities of daily living, mental health, involvement in social life, and the quality of relationships in the environment in which they function [12,14].

A big problem in Poland is the lack of integrity between the medical and social departments that care for elderly patients with frailty syndrome. The medical department takes care of those who need medical help, while the social department takes care of those who live in complex social conditions. It is emphasized that creating a coordinated care model for an elderly patient that includes a primary care physician or geriatrician, a day clinic, inpatient departments, and social care is necessary. No primary health care clinic combines primary health care with outpatient specialist care (dietetics, physiotherapy, psychology) and provides a comprehensive treatment of frail patients in one place [15]. Apart from one-off prevention programs implemented in Poland for older people with frailty syndrome, such as FOCUS, SUN-FRAIL, and Ja Advantage [16], there are no programs aimed at specifying patients in need of appropriate intervention or identifying older people at risk of falling ill due to frailty.

The study aimed to determine the level of satisfaction of health, psychological, environmental, and social needs (1) and to determine the impact of dietary intervention and physical and comprehensive activity (nutritional intervention plus physical activity) on the level of needs satisfaction of older adults with frailty syndrome (FS) (2).

## 2. Materials and Methods

### 2.1. Study Design and Setting

A multicenter experimental study was conducted from May 2017 to December 2017 among family physician patients, students at the University of the Third Age, participants of senior citizens’ clubs, daycare centers, and nursing home residents in Mazowieckie, Małopolskie, Opolskie, and Dolnośląskie voivodeships. To disseminate information about the project and recruit as large a group of subjects as possible, a publicity campaign was conducted among the elderly. The start of the research was preceded by a symposium at which the problem of frailty syndrome was presented. Individuals interested in participating in the study were allowed to make an appointment for a qualifying examination. Patients unable to attend the symposium were provided with comprehensive information about frailty. The study was divided into three phases. Phase 0 (T0)-during the first meeting with patients, the problem was presented by FS. After obtaining consent for the study, patients were qualified for the presence of pre-frail or FS using the Cardiovascular Health Study scale [8].

### 2.2. Interventions

Each patient who qualified for the study received informational materials about FS. Patients self-selected the type of intervention in which they wished to participate. Intervention groups were formed: G1-Diet (patients were instructed on how to write down their daily meals. They had to consider each element of the feed consumed, its size, and the number of fluids consumed. Next, each patient created their 3–4 day meal plan, which a dietitian then analyzed for the amount of protein, vegetables, fiber, and carbohydrates consumed. Each patient received individual and general dietary recommendations for daily use at home. The recommendations included an incorrect element of the diet and also had. Also, they formed several proposed meals and products that can be included in the daily menu. They recommended meal sizes. G2-physical activity (with the help of a physiotherapist, sets of exercises were prepared specifically for older adults with FS. One hour of training included warming up, strengthening, and aerobic and stretching exercises. To perform the complete activities, readily available tools were enough: a mat, a bottle of water, and a chair.

Each patient received a description of the exercises and a CD with recorded exercises to perform at home. The recommended frequency of physical activity was 1 h of exercise twice a week). Patients also had the opportunity to participate in group exercises in the training room, where they could verify the correctness of the practices, G3-comprehensive therapy (nutritional intervention combined with physical activity), G4-control (patients were informed about the health problem, prophylaxis and treatment of TS, received information materials but were not subjected to other interventions. Stage 1 (T1)-3 months after the first examination, the measurements from stage 0 were repeated, and the patients completed the questionnaires and questionnaires. Stage 2 (T2)-After another three months, the measurements from stage 0 were repeated, and the patients completed the questionnaires and questionnaires again. As research shows, the interventions used in our research are one of the most beneficial in preventing and treating weakness syndrome; at the same time, they are simple to perform and apply to elderly patients [17,18,19,20]. 

### 2.3. Participants

In this case, 188 patients were enrolled in the study, including 140 women and 48 men aged 60–94. The inclusion criteria for patients in the study were age over 60 years, diagnosed frailty or prefrailty based on the Fried scale criterion, and consent to participate. The exclusion criteria were lack of patient support to participate in the study, age under 60 years, no diagnosed FS or pre-frail, a somatic condition that prevented full completion of the survey (e.g., visual impairment, severe mental disorder, or another difficulty that prevented participation in the study).

### 2.4. Measurement Tool and Outcome

During the initial interview with patients, qualifications were based on the Cardiovascular Health Study [8]. The scale assesses five aspects that can be used to identify patients with premature or full-blown FS (weight, fatigue, handgrip strength, walking speed and level of physical activity).

#### 2.4.1. Weight

The first was the patient’s unintentional weight loss of approximately 4.5 kg or 5% of body weight. This value is based on available medical records or, if such descriptions are unavailable, on the patient’s subjective assessment. If a patient lost weight, 1 point was assigned.

#### 2.4.2. Fatigue

The second aspect was the assessment of fatigue using the CES-D depression scale (Center for Epidemiological Studies Depression) [21]. The CES-D scale focuses on examining the occurrence of affective symptoms, particularly depressed mood [22]. The CES-D consists of a list of 20 statements that describe the respondent’s well-being or behavior during the past week [23]. Responses to two of the 20 questions were analyzed: “I felt that everything I did require effort” and “I was unable to do anything” (questions 7 and 20). If a patient answered “sometimes or moderately” or “most of the time or all of the time” to at least one of these questions, 1 point was awarded.

#### 2.4.3. Handgrip Strength

The third aspect assessed was handgrip strength, measured with a Saehan pneumatic dynamometer. The dynamometer measures the force of the hand when squeezed with an accuracy of 1 kg. The measurement was performed in a sitting position (the patient’s feet were on the floor, the arms were moved along the body, the elbow was bent 90 degrees, the forearm was in a neutral position, and the wrist was extended between 0 and 30 degrees) [24]. Patients pressed the force gauge three times with each hand, with a one-minute pause between measurements. The highest height was recorded. To determine the patient’s result, BMI (body mass index) was also determined using the following formula: BMI = body weight in kg/body height in m^2^ [25]. Based on the obtained measurements, the range of parameters in which the patient’s results fit was determined [8]:


**Women:**

**Men:**
If:If:BMI ≤ 23 ≤ 17.0 kgBMI ≤ 24 ≤ 29 kgBMI 23.1–26 ≤ 17.3 kgBMI 24.1–26 ≤ 30 kgBMI 26.1–28 ≤ 18 kgBMI 26.1–28 ≤ 30 kgBMI > 29 ≤ 21 kgBMI > 28 ≤ 32 kgIt was granted to the patient. 1 point, And if the measurement is >21 kg, the patient receives 0 points.It was granted to the patient 1 point, And if the measurement is >32 kg, the patient receives 0 points.

#### 2.4.4. Walking Speed

The fourth aspect assessed was the evaluation of the patient’s walking speed over 4.6 m. The patient walks the specified distance of 4.6 m with command lines. To analyze the obtained result, the patient’s height was also measured with an accuracy of 0.5 cm. Based on the obtained results, 1 or 0 points were assigned [8]:


**Women:**

**Men:**
If:If:height ≤ 159 cm ≥ 7 sheight ≤ 173 cm ≥ 7 sheight > 159 cm ≥ 6 sheight > 173 cm ≥ 6 sIt was granted to the patient. 1 point, and if he did not meet the above criteria: 0 pointsIt was granted to the patient 1 point, and if he did not meet the above criteria: 0 points

#### 2.4.5. Level of Physical Activity

The last instrument used on the Fried scale was the IPAQ (International Physical Activity Questionnaire), which assesses the level of physical activity in the past seven days about daily life, work, and leisure. Each exercise type is set in MET-min/week. The patient falls into three activity categories: high, sufficient, and insufficient [26]. Patients who achieved an inadequate level of physical activity received one point.

Patients who scored 1 or 2 out of 5 possible points in the above five aspects were classified as pre-frail, and patients who scored 3 to 5 points were classified as FS [8]. Patients who scored 0 points were not eligible for the study.

Qualified patients completed the original questionnaire, including age, sex, education, marital status, place of residence, employment, financial situation, and chronic diseases.

#### 2.4.6. Needs Evaluation

The patient’s level of need satisfaction was determined using Camberwell’s Modified Short Needs Assessment [27]. This questionnaire is a simple instrument that focuses on 22 problem areas of chronic somatic patients without mental disorders—internal consistency of the questionnaire: Cronbach’s alpha: 0.82. Camberwell index (IC) was calculated based on 24 questions. For its calculation, the formula M/N was used (M-number of questions defining the satisfied need, N-number of questions to which the patient responded). The higher the index, the higher the degree of need satisfaction [27].

### 2.5. Compliance with Ethical Standards

The study was conducted by the requirements of the Declaration of Helsinki and the principles of Good Clinical Practice. The Bioethics Committee approved the study of the Medical University of Wroclaw (No. KB 211/2017). Before the study, each participant was informed about the purpose and expected benefits of the study. Patients were told that their participation in the study was voluntary, and they were assured they could withdraw from the study at any time. Informed consent signed by the patients was a prerequisite for participation in the study.

### 2.6. Statistical Methods

The following values were calculated for the quantitative variables in this study: Mean (M), standard deviation (SD), median (Me), minimum (min), and maximum (max). For the quantitative variables in this study, the normality of the distribution this study’s quantitative variables distribution’s normality was examined using the Shapiro-Wilk test. The Shapiro-Wilk test tested the null hypothesis that the variable has a normal distribution versus the alternative that the variable does not have a normal distribution. It was assumed that the significance level was α = 0.05. To test the central hypothesis, the nonparametric Quade test was used, which is used for successive measurements on the same patient, the so-called test for multiple sizes. Correspondence analysis was used to determine the most frequently associated variables with T0, T1, and T2 and with the groups G1, G2, G3, and G4. The statistical package R 3.1.3 for Mac OS X 10.11.5 and the software Exel 2013 were used to perform the calculations. The authors of the article carried out data analysis.

## 3. Results

### 3.1. Descriptive Data

In all groups, the majority were women, respondents with a low or medium level of education (41.7%-low, 41.7%-medium), and in relationships (98%). Most patients lived in rural areas (52.5%). 78.9% of respondents were retired or not working and described their financial situation as quite good (71.2%) (Table 1).

### 3.2. Main Results

Statistical analysis showed that the degree of need satisfaction in food group G1 did not depend on the time of measurement (*p* = 0.129). The degree of need satisfaction decreased slightly after three months and increased six months after the start of the intervention to a value higher than before the beginning of the nutrition intervention, but it was not statistically significant (*p* = 0.129) (Table 2).

The distribution of the level of need satisfaction in the G2 group was dependent on the time of measurement (*p* = 0.019). Statistically significant was the difference between the terms T0 and T2 (p02 = 0.018) (Me = 0.88 (min-max. 0.38–1.00) (*p* < 0.001) and Me = 0.93 (min-max. 0.65–1.00) (*p* = 0.005) The differences between T0, T1 and between T1 and T2 were not statistically significant (p01 = 0.084, p12 = 0.383) Me = 0.88 (min-max 0.38–1.00) (*p* < 0.001), Me = 0.91 (min-max 0.61–1.00) (*p* = 0.001) and Me = 0.93 (min-max 0.65–1.00) (*p* = 0.005) (Table 3).

In the G3 complex group, the distribution of the Camberwell Needs Index was dependent on the date of measurement (*p* = 0.007). The differences between the remaining term T0a were statistically significant (p01 = 0.009, p02 = 0.024). A statistically significant increase in the value of the variable was observed in the last two measurements: Me = 0.83 (min-max 0.38–1.00) (*p* = 0.007), Me = 0.89 (min-max 0.59 -1.00) (*p* = 0.028) and Me = 0.88 (min-max 0.35–1.00) (*p* < 0.001), but the decrease between measurements T1 and T2 was not statistically significant (p12 = 0.548) (Table 4).

In control group G4, the degree of need satisfaction depended on the time of measurement (*p* = 0.016). There were statistically significant differences between T0 and the other measurement time points (p01 = 0.037, p02 = 0.022). A statistically significant increase in the mean value of the variable was observed in the following time periods: M = 0.67 SD = 0.19 (*p* = 0.130), M = 0.70 SD = 0.19 (*p* = 0.033), M = 0.78 SD = 0.17 (*p* = 0.016), but the increase between T1 and T2 data was not statistically significant (*p* = 0.651) (Table 5).

**Figure 1:**
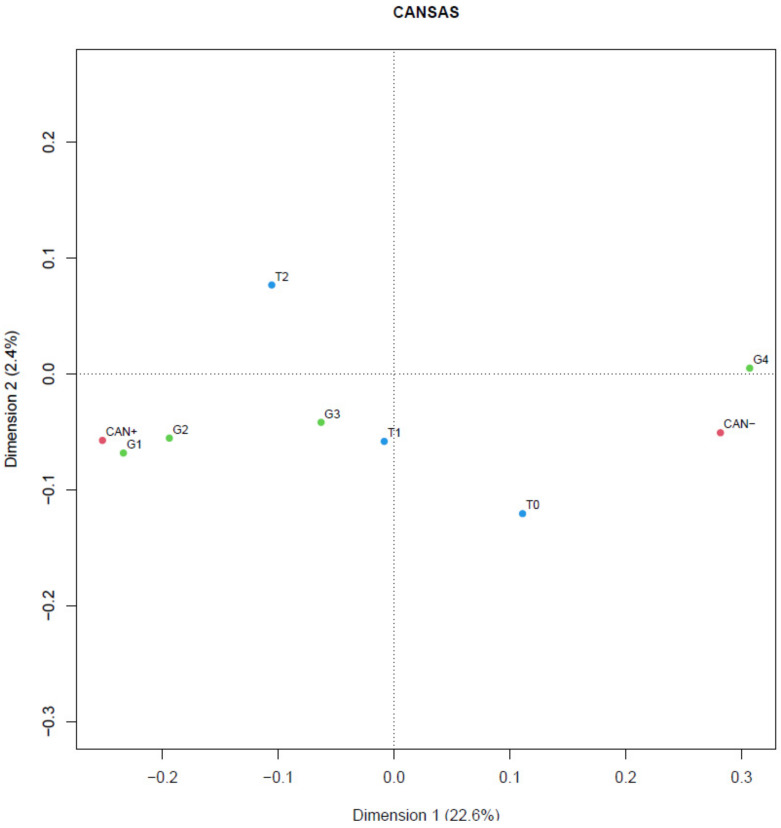
Level of satisfaction of needs in therapeutic-time subgroups-correspondence analysis. Source: own study. Legend: T0-examination date before the start of the intervention, T1-examination date three months after the start of research and intervention, T2-examination date 3 months after the start of research and intervention, G1-group. nutritional, G2-group physical activity, G3-group comprehensive, G4-group control, CAN + -short needs assessment Camberwell ≥mediana, CAN− -short needs assessment Camberwell < median.

In the studied groups of patients, a higher level of need satisfaction (CAN +) was observed in groups G1, G2, and G3 and during T1 and T2, and a lower level of need satisfaction (CAN-) was observed in group G4 and T0 (Figure 1).

## 4. Discussion

### 4.1. Key Results and Interpretation

Increasingly unmet needs in patients increase the risk of hospitalization threefold compared to patients with high levels of need satisfaction [28]. Based on studies conducted in 11 highly developed countries, patients with FS were found to have high levels of needs, referred to as high-need patients. These were patients aged 65 and older with at least three chronic diseases or dysfunctions of daily living. These patients were more likely to use health care services, less financially well off, and less able to coordinate their care than other older patients [29]. Unmet needs interfere with daily living, hinder acute and chronic inflammation treatment, and lead to further health problems and complications [30]. Assessing the degree of need satisfaction of elderly patients with vulnerability FS and the factors influencing it is an essential element of care planning. Older people are characterized by more significant heterogeneity over time, which is also a consequence of increasingly differentiated needs. Three conditions are particularly emphasized in care: physical, psychosocial, and spiritual [30].

The results of our own study showed that the highest satisfaction of needs among patients interviewed before the intervention was reported by patients in the nutrition group (G1) (M = 0.86, SD = 0.14, *p* < 0.001), followed by the physical activity group (G2) (M = 0.84 SD = 0.13, *p* < 0.001) and the comprehensive group (G3) (M = 0.80 SD = 0.15, *p* = 0.007), and the lowest level was observed in the control group (G4) (M = 0.67 SD = 0.19, *p* = 0.130). Patients in the intervention groups had a higher Camberwell index than in Kurpas’ studies conducted among 3602 chronically ill patients (M = 0.77 SD = 0.17) [19]. In the Szwamel study, patients in primary care and hospital emergency departments achieved similar mean scores to those in the intervention groups: 0.80 and 0.75, respectively (*p* = 0.008) [31]. The control group differed significantly from the results obtained in other studies.

A statistically significant increase in the level of need satisfaction was demonstrated in the intervention groups with physical activity (G2 and G3). It should be emphasized that there was also a significant increase in the control group G4, which was the highest among the studied groups despite the absence of intervention. It is possible that this is because the initial level of need satisfaction in this group was much lower than in the other groups and eventually turned out to be lower than the level of need satisfaction in the comprehensive group before the intervention. A similar conclusion can be drawn for group G1, in which an increase in the level of need satisfaction was observed. Still, it was not statistically significant and represented the smallest increase among the groups studied. However, in this group, the baseline level of need satisfaction was the highest. As the author’s research results indicate, among the selected forms of intervention, the increase in need satisfaction is most effectively influenced by the initiation of physical activity. The results of the author’s research coincide with the analysis of Kubińska, who studied the health needs for physical activity in the elderly with 221 people aged 60–90 years. She found that the seniors interviewed fulfilled the requirements for family, prevention, exercise, recreation, and tourist activity through physical activity [32].

It is also worth paying attention to the gender ratio among project participants. Most of the study participants were women (74%), which is also observed in the studies by Ng et al. [19] and da Silva et al. [33]. Research shows that women are more willing to participate in projects and undergo interventions. It is worthwhile to pay more attention to men in clinical procedures so that they are more willing to participate in interventions and work to improve their health.

### 4.2. Strengths of the Study

The study included three intervention groups and one control group. The sensitive Fried scale was used to identify patients with FS. A positive aspect of the study is the diversity of the places of residence of the patients studied (no less than four provinces), both urban and rural, participants from universities of the Third Age, senior clubs, nursing home patients, and primary health care patients.

### 4.3. Limitations

A limitation of the study is the size of the groups that participated in the interventions. In this case, 188 individuals participated in the study. This is too small a group to generalize the results of our research to the entire population of older people in Poland. Another weakness of the study is its time limitation. The 6-month analysis is too short to clearly define the most beneficial forms of intervention. Another limiting element was the timing of the intervention. The third phase took place in the second half of December, during the vacation season, when respondents were leaving, staying with relatives in other homes, and did not have time to meet to take the necessary measurements and complete extensive questionnaires. Another limitation is the time period in which the tests are administered. There was a loss of participants in the study. This was due to the deterioration of their health, which made it impossible for them to continue their participation in the study survey (hospitalizations, surgeries), the decreasing motivation to carry out the intervention, and the unfortunate timing of the third measurement, which took place around Christmas, when some of the participants were traveling to their families, and the fact that they did not find time to review the extensive survey, which took about 1 h.

## 5. Conclusions

The introduction of physical activity and exercise in combination with dietary changes most effectively influences the increase in the satisfaction level of the needs of elderly patients with FS. The research findings could be used to develop monitoring, prevention, and education programs for older patients with frailty syndrome. They may facilitate the development of care plans that address the met and unmet needs of patients in their home environment.

## Figures and Tables

**Table 1:** Sociodemographic data of patients in the groups G1, G2, G3 and G4.

Variable		Group				Total
		G1	G2	G3	G4	
Gender	Women	30 (75%)	26 (61.9%)	36 (80%)	48 (76.2%)	140 (74%)
	Men	10 (25%)	16 (38.1%)	9 (20%)	15 (23.8%)	50 (26%)
	total	40 (100%)	42 (100%)	45 (100%)	63 (100%)	190 (100%)
Age (years)	n	38	42	45	60	185
	M ± SD	69.87 ± 6.17	69.38 ± 6.66	70.80 ± 6.09	79.13 ± 9.04	-
	Me	69.50	67.00	70.00	79.50	-
	min.–max.	61.00–91.00	60.00–85.00	60.00–85.00	60.00–94.00	60.00–94.00
Education	low (elementary, middle school)	12 (30%)	18 (42.9%)	16 (35.6%)	32 (53.3%)	78 (41.7%)
	secondary (secondary or post-secondary)	21 (52.5%)	16 (37.1%)	18 (40%)	23 (38.3%)	78 (41.7%)
	high (higher)	7 (17.5%)	8 (19%)	11 (24.4%)	5 (8.3%)	31(16.6%)
	total	40 (100%)	42 (100%)	45 (100%)	60 (100%)	187 (100%)
Marital status	bachelor/maiden	3 (7.5%)	3 (7.1%)	3 (6.7%)	6 (9.8%)	15 (0.08%)
	married	26 (65%)	29 (69%)	27 (60%)	16 (26.2%)	98 (52.1%)
	widower/widow	11 (27.5%)	9 (21.4%)	14 (31.1%)	36 (59%)	70 (37.2%)
	divorced/divorced	0 (0%)	1 (2.4%)	1 (2.2%)	3 (4.9%)	4 (2.2%)
	together	40 (100%)	42 (100%)	45 (100%)	61 (100%)	188 (100%)
In a steady relationship	yes	27 (67.5%)	28 (70%)	26 (61.9%)	19 (31.1%)	100(53.2%)
	no	13 (32.5%)	12 (30%)	16 (37.1%)	42 (68.9%)	83(45.4%)
	total	40 (100%)	40 (100%)	42 (100%)	61 (100%)	183 (100%)
With whom he lives	alone	11 (28.2%)	9 (21.4%)	8 (17.8%)	15 (24.6%)	43 (22.9%)
	only with a husband/wife, partner/partner	14 (35.9%)	14 (33.3%)	14 (31.1%)	9 (14.8%)	78 (41.7%)
	with family	14 (35.9%)	19 (45.2%)	23 (51.1%)	22 (36.1%)	187 (100%)
	in the nursing home	0 (0%)	0 (0%)	0 (0%)	15 (24,6%)	15 (8%)
	total	39 (100%)	42 (100%)	45 (100%)	61 (100%)	187 (100%)
Place of living	in a large city (>100,000 inhabitants)	4 (10.3%)	4 (9.5%)	13 (30.2%)	10 (17.2%)	31 (17.3%)
	in a medium-sized city (20,000–100,000 inhabitants)	15 (38.5%)	12 (28.6%)	7 (16.3%)	13 (22.4%)	47 (26.3%)
	in a small town (under 20,000 inhabitants)	1 (2.6%)	6 (14.3%)	3 (7%)	0 (0%)	10 (5.6%)
	In the countryside	19 (48.7%)	20 (47.6%)	20 (46.5%)	35 (60.3%)	94 (52.5%)
	total	39 (100%)	42 (100%)	43 (100%)	58 (100%)	179 (100%)
Work status	I am working	3 (7.5%)	5 (12.2%)	2 (4.4%)	1 (1.6%)	11 (5.9%)
	I do not work, I am retired	29 (72.5%)	31 (75.6%)	36 (80%)	50 (82%)	146 (78.9%)
	I am not working, I am unemployed	0 (0%)	1 (9.8%)	0 (0%)	1 (1.6%)	2 (1.1%)
	I am retired and working	8 (20%)	4 (9.8%)	7 (15.6%)	9 (16.1%)	28 (15.1%)
	total	40 (100%)	41 (100%)	43 (100%)	61 (100%)	185 (100%)
Financial situation	very good	0 (0%)	1 (2.5%)	0 (0%)	9 (16.1%)	10 (5.6%)
	pretty dobra	33 (89.2%)	35 (87.5%)	31 (70.5%)	27 (48.2%)	126 (71.2%)
	bad	4 (10.8%)	4 (10%)	13 (29.5%)	20 (35.7%)	41 (23.2%)
	total	37 (100%)	40 (100%)	44 (100%)	56 (100%)	177 (100%)
Help in household chores	yes	5 (12.5%)	3 (7.1%)	6 (13.6%)	27 (43.5%)	41 (21.8%)
	no	35 (87.5%)	39 (92.9%)	38 (86.4%)	35 (56.5%)	147 (78.2%)
	total	40 (100%)	42 (100%)	44 (100%)	62 (100%)	188 (100%)

**Table 2:** The level of satisfaction of the needs in the G1 food group during T0, T1, T2.

Variable	Time	n	M	SD	Me	Min.	Max.	*pSW*	*pQ*	*diff*	*meandiff*	*Quademcomp*		
**Index** **Camberwell**	**T0**	40	0.86	0.14	0.90	0.31	1.00	<0.001	0.129	**n**	23	**p**	**T0**	**T1**
	**T1**	36	0.85	0.14	0.89	0.35	1.00	<0.001		**T1−T0**	0.02	**T1**	0.271	-
	**T2**	23	0.90	0.09	0.93	0.61	1.00	0.002		**T2−T1**	0.01	**T2**	0.143	0.510

**Legend: T0**-time of the first examination of patients, before the start of the intervention, **T1**-time of the second examination of patients, 3 months from the start of the intervention, **T2**-time of the third examination of patients, 6 months from the start of the intervention, **n**- number of patients; **M** average; **SD**-standard deviation; **Me**-median; **min.**-minimum; **max**.- maximum, ***pSW***-calculated significance level of the Shapiro-Wilk test checking the hypothesis that the variable is normal distribution, ***pQ***-calculated significance level of the Quade test checking the hypothesis that the distribution of the variable in repetitive measurements T0, T1 and T2 is the same, ***diff***-difference, ***meandiff***-mean difference between the results of the terms (T1 and T0, T2 and T1), ***Quademcomp* p**-calculated significance levels for the Quade’s p test of multiple comparisons for each pair of terms T0, T1 and T2.

**Table 3:** The level of satisfaction of needs in the G2 physical activity group during T0, T1, T2.

Variable	Time	n	M	SD	Me	Min.	Max.	*pSW*	*pQ*	*diff*	*meandiff*	*Quademcomp*		
**Index** **Camberwell**	**T0**	42	0.84	0.13	0.88	0.38	1.00	<0.001	**0.019**	**n**	24	**p**	**T0**	**T1**
	**T1**	34	0.89	0.10	0.91	0.61	1.00	0.001		**T1−T0**	0.05	**T1**	0.084	-
	**T2**	26	0.90	0.09	0.93	0.65	1.00	0.005		**T2−T1**	0.02	**T2**	0.018	0.383

**Legend: T0**-time of the first examination of patients, before the start of the intervention, **T1**-time of the second examination of patients, three months from the start of the intervention, **T2**-time of the third examination of patients, six months from the beginning of the intervention, **n**-number of patients; **M**-average; **SD**-standard deviation; **Me**-median; **min.**-minimum; **max**.-maximum, ***pSW***-calculated significance level of the Shapiro-Wilk test checking the hypothesis that the variable is normal distribution, ***pQ***-calculated significance level of the Quade test checking the hypothesis that the distribution of the variable in repetitive measurements T0, T1 and T2 is the same, ***diff***-difference, ***meandiff***-mean the difference between the results of the terms (T1 and T0, T2 and T1), ***Quademcomp* p**-calculated significance levels for the Quade’s p test of multiple comparisons for each pair of terms T0, T1 and T2.

**Table 4:** The level of satisfaction of needs in the complex group G3 at the time T0, T1, T2.

Variable	Time	n	M	SD	Me	Min.	Max.	*pSW*	*pQ*	*diff*	*meandiff*	*Quademcomp*		
**Index** **Camberwell**	**T0**	45	0.80	0.15	0.83	0.38	1.00	0.007	**0.007**	**n**	29	**p**	**T0**	**T1**
	**T1**	32	0.87	0.11	0.89	0.59	1.00	0.028		**T1−T0**	0.07	**T1**	0.009	-
	**T2**	32	0.85	0.14	0.88	0.35	1.00	<0.001		**T2−T1**	−0.03	**T2**	0.024	0.548

**Legend: T0**-time of the first examination of patients, before the start of the intervention, **T1**-time of the second examination of patients, 3 months from the start of the intervention, **T2**-time of the third examination of patients, 6 months from the start of the intervention, **n**-number of patients; **M**-average; **SD**-standard deviation; **Me**-median; **min.**-minimum; **max**.-maximum, ***pSW***-calculated significance level of the Shapiro-Wilk test checking the hypothesis that the variable is normal distribution, ***pQ***-calculated significance level of the Quade test checking the hypothesis that the distribution of the variable in repetitive measurements T0, T1 and T2 is the same, ***diff***-difference, ***meandiff***-mean difference between the results of the terms (T1 and T0, T2 and T1), ***Quademcomp* p**-calculated significance levels for the Quade’s p test of multiple comparisons for each pair of terms T0, T1 and T2.

**Table 5:** The level of satisfaction of needs in the G4 control group at the time T0, T1, T2.

Variable	Time	n	M	SD	Me	Min.	Max.	*pSW*	*pQ*	*diff*	*deandiff*	*Quademcomp*		
**Index** **Camberwell**	**T0**	60	0.67	0.19	0.67	0.29	1.00	0.130	**0.016**	**n**	32	**p**	**T0**	**T1**
	**T1**	60	0.70	0.19	0.70	0.35	1.00	0.033		**T1−T0**	0.08	**T1**	0.037	-
	**T2**	38	0.78	0.17	0.80	0.33	1.00	0.016		**T2−T1**	<0.001	**T2**	0.022	0.651

**Legend: T0**-time of the first examination of patients, before the start of the intervention, **T1**-time of the second examination of patients, 3 months from the start of the intervention, **T2**-time of the third examination of patients, 6 months from the start of the intervention, **n**-number of patients; **M**-average; **SD**-standard deviation; **Me**-median; **min.**-minimum; **max**.-maximum, ***pSW***-calculated significance level of the Shapiro-Wilk test checking the hypothesis that the variable is normal distribution, ***pQ***-calculated significance level of the Quade test checking the hypothesis that the distribution of the variable in repetitive measurements T0, T1 and T2 is the same, ***diff***-difference, ***meandiff***-mean difference between the results of the terms (T1 and T0, T2 and T1), ***Quademcomp* p**-calculated significance levels for the Quade’s p test of multiple comparisons for each pair of terms T0, T1 and T2.

## Data Availability

The data presented in this study are available on request from the corresponding author.
